# Intentional Undercorrection by Implantation of Posterior Chamber Phakic Intraocular Lens With A Central Hole (Hole ICL) For Early Presbyopia

**DOI:** 10.1155/2018/6158520

**Published:** 2018-06-10

**Authors:** Masahide Takahashi, Kazutaka Kamiya, Nobuyuki Shoji, Sayaka Kato, Akihito Igarashi, Kimiya Shimizu

**Affiliations:** ^1^Department of Ophthalmology, Kitasato University, Kanagawa, Japan; ^2^School of Allied Health Sciences, Kitasato University, Kanagawa, Japan; ^3^Department of Ophthalmology, Sanno Hospital, Tokyo, Japan

## Abstract

**Purpose:**

To assess visual performance at near to far distances in early presbyopic patients with undercorrection by implantation of an ICL with a central hole (hole ICL).

**Methods:**

This prospective study evaluated forty-two eyes of 21 early presbyopic patients (age, 40 to 53 years) with spherical equivalents of -7.37 ± 3.18 D [mean ± standard deviation] who underwent hole ICL implantation and whose targeted refraction was set at slight myopia (-0.61 ± 0.28 D) for both eyes. We assessed the safety, efficacy at near to far distances, predictability, and adverse events of the surgery, during the 6-month observation period.

**Results:**

Corrected distance visual acuity did not improve significantly, from -0.17 ± 0.07 preoperatively to -0.19 ± 0.08 logMAR postoperatively (p=0.066, Wilcoxon signed-rank test). Uncorrected distance visual acuity was significantly improved from 1.30 ± 0.24 preoperatively to -0.03 ± 0.20 logMAR postoperatively (p<0.001). The mean binocular visual acuity was 0.02 logMAR or better at all distances (5.0, 3.0, 2.0, 1.0, 0.7, 0.5, and 0.3 m). All eyes were within ± 0.5 D of the targeted correction. Neither cataract formation, significant intraocular pressure rise, nor other vision-threatening complications occurred in any case during the 6-month observation period.

**Conclusions:**

Our pilot study showed that intentional undercorrection by hole ICL implantation for early presbyopia was safe with predictable refractive results and provided good binocular vision at near to far distances, without developing cataract, suggesting its viability as a surgical presbyopic treatment for such patients.

## 1. Introduction

The Visian Implantable Collamer Lens (ICL), a posterior chamber phakic intraocular lens, has been demonstrated to offer effective correction of moderate to high ametropia over a long period of time [1–6]. However, this surgery necessitates preoperative laser iridotomies or intraoperative peripheral iridectomy to prevent the occurrence of pupillary block at all times. Furthermore, the surgeons are still concerned about the possible risk of cataract formation especially in older patients. A new ICL with a central hole (hole ICL) has been developed in order to resolve such problems [7, 8]. We previously showed, from clinical and optical viewpoints, that visual performance after ICL implantation was significantly better than that after wavefront-guided laser in situ keratomileusis (wfg-LASIK), even in the presence of low to moderate myopic defocus [9]. Accordingly, we hypothesize that undercorrection by hole ICL implantation contributes to obtaining better visual performance at near distance and clinically acceptable visual performance at far distance, especially for early presbyopic subjects, without developing cataract. The goal of our study is to prospectively assess the safety, efficacy including binocular visual performance at near to far distances, and predictability of intentionally undercorrected ICL implantation for the correction of moderate to high ametropia in early presbyopic subjects.

## 2. Materials and Methods

### 2.1. Study Population

The protocol was registered with the University Hospital Medical Information Network Clinical Trial Registry (000025892). Forty-two eyes of 21 consecutive patients (10 men and 11 women), who underwent implantation of the posterior chamber phakic intraocular lens with a 0.36-mm central hole (hole ICL, KS-Aquaport™; STAAR Surgical, Nidau, Switzerland) for the correction of moderate to high myopia and whose age was 40 years or more, were included in this prospective study. Eyes with keratoconus were excluded from the study by using the screening test of Placido disk videokeratography (TMS-2, Tomey, Nagoya, Japan). We selected toric ICL implantation in eyes with manifest astigmatism of 1.5 diopters (D) or more and nontoric ICL implantation in eyes with that of less than 1.5 D. Preoperatively and 6 months postoperatively, we measured the logarithm of the minimal angle of resolution (logMAR) of monocular distance visual acuity (UDVA) and binocular uncorrected visual acuity at 5.0, 3.0, 2.0, 1.0, 0.7, 0.5, and 0.3-m distances, logMAR corrected distance visual acuity (CDVA), and manifest refraction (spherical equivalent and cylinder), in addition to the usual slit-lamp biomicroscopic and funduscopic examinations. Binocular visual acuity measurements were performed at 5.0, 3.0, 2.0, 1.0, 0.7, 0.5, and 0.3 m with best correction using an all-distance vision tester (AS-15, Kowa, Tokyo, Japan). The patient satisfaction for overall visual performance was assessed at 6 months postoperatively, according to the visual analog scale in a range from 0 (very dissatisfied) to 10 (very satisfied). The study was approved by the Institutional Review Board at Kitasato University School of Medicine and followed the tenets of the Declaration of Helsinki. Informed consent was obtained from all patients after explanation of the nature and possible consequences of the study.

### 2.2. Lens Power and Size Calculation

The ICL power was determined by the online calculator of the manufacturer (STAAR Surgical) using a modified vertex formula. We intentionally selected undercorrection of approximately -0.50 to –1.50 D in both eyes as the target refraction, which was individually determined with visual simulation with contact lenses wearing in each patient, based on patient age and patient preference for vision. The ICL size was also chosen by the manufacturer on the basis of the horizontal corneal diameter and the anterior chamber depth measured with scanning-slit topography (Orbscan IIz, Bausch & Lomb, Rochester, NY, USA).

### 2.3. Surgical Procedure

The surgical procedures in our institution were comprised as follows, as described previously [7, 8]. In brief, after topical administration of dilating and anesthetic agents, a model V4c ICL was inserted through a 3-mm temporal corneal incision with the use of a viscosurgical device into the anterior chamber. The ICL was placed in the posterior chamber, the viscosurgical device was fully washed out with balanced salt solution, and then a miotic agent was instilled. Postoperatively, steroidal and antibiotic medications were administered topically 4 times daily for 2 weeks, the dose being reduced gradually thereafter.

### 2.4. Statistical Analysis

All statistical analyses were conducted by commercially available statistical software (BellCurve for Excel, Social Survey Research Information Co, Ltd., Tokyo, Japan). The normality of all data samples was first checked by the Shapiro-Wilk test. Since the data did not fulfill the criteria for normal distribution, the Wilcoxon signed-rank test was used for statistical analysis to compare the pre- and postsurgical data. Unless otherwise indicated, the results are expressed as mean ± standard deviation, and a value of p<0.05 was considered statistically significant.

## 3. Results

### 3.1. Study Population

The preoperative and postoperative demographics are listed in Table 1. The targeted refraction was -0.61 ± 0.28 D. No intraoperative complications occurred, and no eyes were lost during the 6-month follow-up, in the study population.

### 3.2. Safety Outcomes

**Figure 1:**
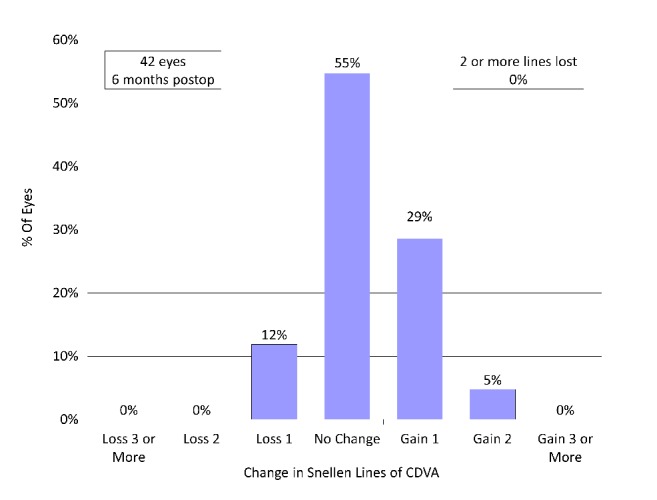
Changes in corrected distance visual acuity (CDVA) after hole implantable collamer lens (hole ICL) implantation.

CDVA did not improve significantly, from -0.17 ± 0.07 preoperatively to -0.19 ± 0.08 logMAR postoperatively (p=0.066, Wilcoxon signed-rank test). Twenty-three eyes (55%) showed no change in CDVA, 12 eyes (29%) gained 1 line, 2 eyes (5%) gained 2 lines, and 5 eyes (12%) lost 1 line, 6 months postoperatively (Figure 1).

### 3.3. Efficacy Outcomes at Near to Far Distances

**Figure 2:**
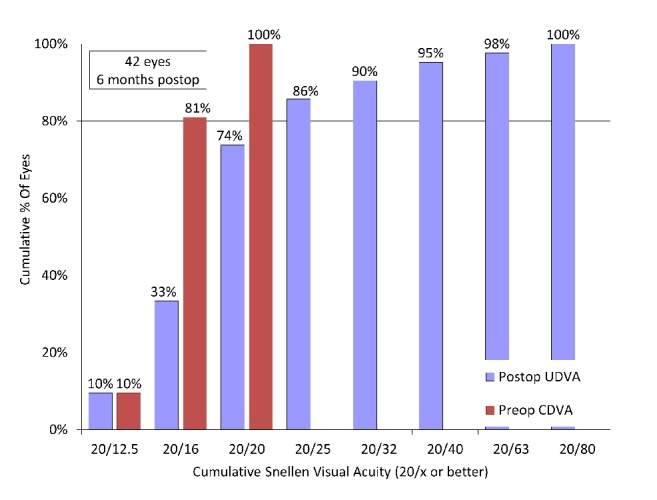
Cumulative percentages of eyes attaining specified levels of postoperative uncorrected distance visual acuity (UDVA) compared to the cumulative percentages of eyes attaining specified levels of  .preoperative corrected distance visual acuity (CDVA) after hole implantable collamer lens (hole ICL) implantation.

**Figure 3:**
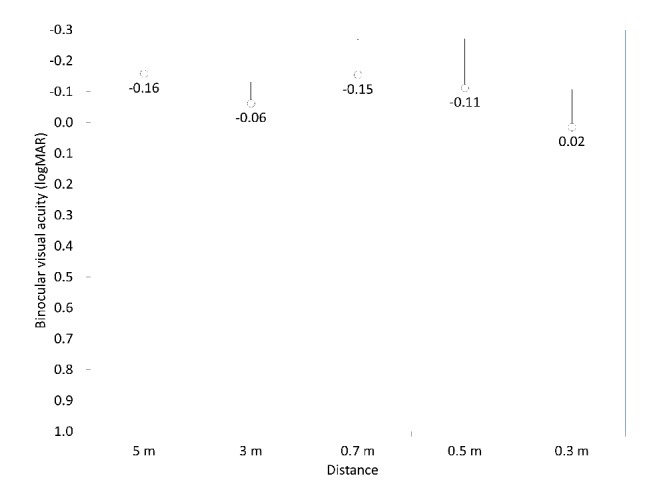
Uncorrected visual acuity at near to far distances after hole implantable collamer lens (hole ICL) implantation.

UDVA was significantly improved from 1.30 ± 0.24 preoperatively to -0.03 ± 0.20 logMAR postoperatively (p<0.001, Wilcoxon signed-rank test). The cumulative percentages of eyes attaining specified cumulative levels of UDVA are listed in Figure 2. The postoperative binocular uncorrected visual acuity at 5.0, 3.0, 2.0, 1.0, 0.7, 0.5, and 0.3 m distances are listed in Figure 3. The mean binocular visual acuity was 0.02 logMAR or better at all distances.

### 3.4. Predictability

**Figure 4:**
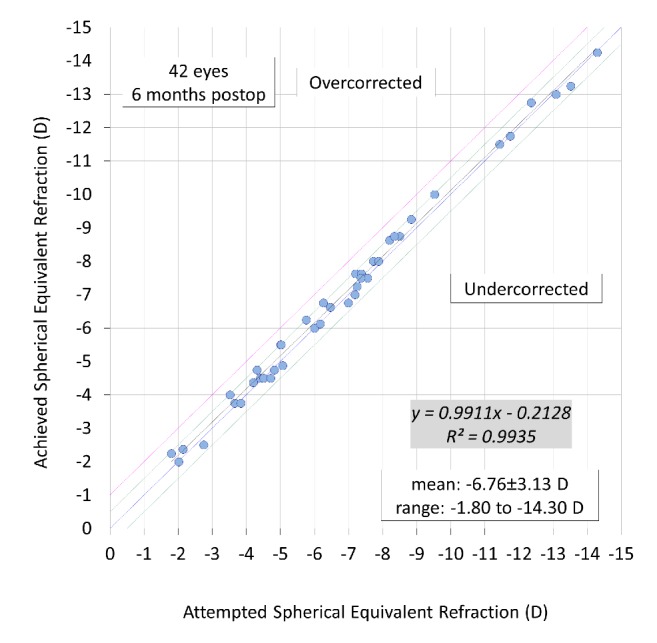
A scatter plot of attempted versus achieved correction (spherical equivalent) after hole implantable collamer lens (hole ICL) implantation.

**Figure 5:**
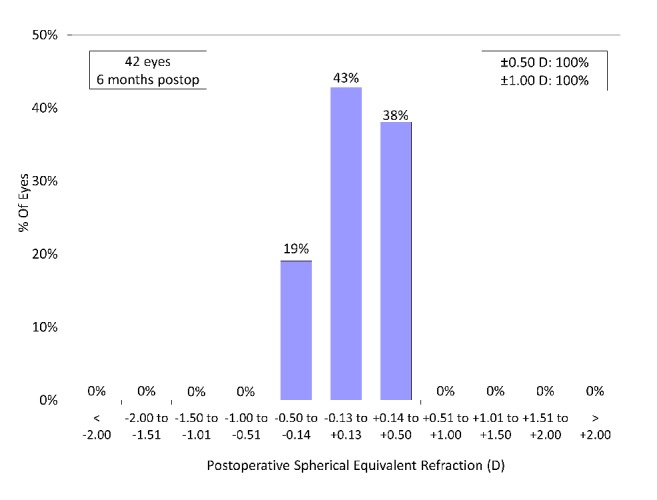
Spherical equivalent refractive accuracy after hole implantable collamer lens (hole ICL) implantation.

**Figure 6:**
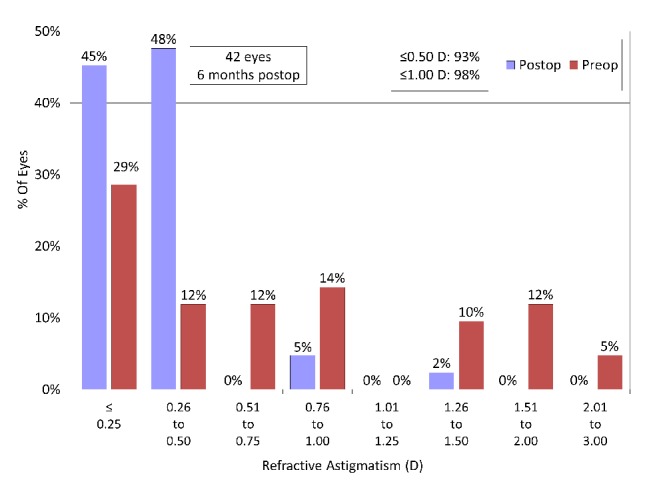
Preoperative and postoperative refractive astigmatism after hole implantable collamer lens (hole ICL) implantation.

A scatter plot of the attempted versus the achieved manifest spherical equivalent correction, the spherical equivalent refractive accuracy, and the preoperative and postoperative refractive astigmatism are listed in Figures 4–6. All eyes were within ± 0.5 D of the targeted correction.

### 3.5. Patient Satisfaction

The postoperative satisfaction score was 8.2 ± 1.1 (range: 7 to 10). All patients have been satisfied with overall visual performance after surgery.

### 3.6. Adverse Events

Eight (19%) of 42 eyes developed glare or halo in the early postoperative period, but symptoms were mild and no secondary intervention was required. Neither cataract formation, significant intraocular pressure rise, pigment dispersion glaucoma, pupillary block, severe subjective symptoms such as glare or halo, nor any other vision-threatening complications occurred at any time in this series.

## 4. Discussion

In the present study, our preliminary results of undercorrected correction by hole ICL implantation were favorable in all measures of safety, efficacy (especially binocular vision), and predictability, without developing cataract, when used for the correction of moderate to high ametropia. We previously demonstrated that visual performance in ICL-implanted eyes was significantly better than that in post-LASIK eyes, even in the presence of low to moderate myopia [9]. Therefore, we hypothesize that intentional undercorrection by ICL implantation may be useful for obtaining better visual performance at near to far distances, even in early presbyopic subjects. Our results also revealed that binocular visual acuity at all distances was overall good even in early presbyopic patients, without cataract formation, suggesting its viability as a surgical option for the presbyopic treatment of such eyes.

With regard to the safety, efficacy, and predictability of this surgery, hole ICL implantation was safe and effective and provided predictable results for the correction of moderate to high myopia, findings which were in agreement with previous studies [7, 8]. Although monocular UDVA in this study was not very excellent as that in previous studies, since the targeted refraction was set at slight myopia, we assume that binocular visual performance at near to far distances was overall good, and clinically acceptable, for early presbyopic subjects.

With regard to the adverse events of this surgery, it still remains unclear whether hole ICL is effective for the suppression of cataract formation over a long period of time in these presbyopic patients. Gonvers et al. [10] stated that the incidences of ICL-induced cataract in patients of 40 years of age or less and of 41 or over were 14% and 37%, respectively, indicating that ICL-induced cataract develops more frequently in older patients than in younger patients. It has been also demonstrated by Lackner et al. [11] and Sarikkola et al. [12] that ages of 50 years or higher and 45 years or higher, respectively, were risk factors for cataract development after ICL implantation. Fujisawa et al. [13] showed that a decrease in accommodation with aging may influence the circulation of the aqueous humor, resulting in a higher incidence of cataract formation in ICL-implanted eyes. Guber et al. [14] demonstrated that the rate of lens opacity development was 54.8% at 10 years after ICL implantation. Although we accept that 6-month follow-up in a small number of the patients is insufficient to detect rare complications, we believe that hole ICL holds promise for the suppression of cataract formation, presumably due to the improvement of the circulation of the aqueous humor to the anterior surface of the crystalline lens, in early presbyopic patients.

We recently showed monovision by hole ICL implantation is beneficial for acquiring overall good binocular visual performance at all distances in early presbyopic subjects [15]. Although monovision is not tolerable in all patients, it is also one of the viable surgical options for the correction of moderate to high ametropia in early presbyopic subjects. At present, we individually selected intentional undercorrection or monovision, based on the preoperative optical simulation in each patient.

This study is burdened with at least three limitations. Firstly, the maximum follow-up period was set at 6 postoperative months. Considering that a myopic shift due to nuclear sclerosis of the crystalline lens or elongation of the axial length can occur in high myopic eyes, it may result in a worsening distance visual acuity. Although it is unlikely that the refractive and visual outcomes were markedly changed in the late postoperative period, since it is known that this surgical technique provided stable refractive outcomes in previous studies [1–6], more prolonged and careful observation is still necessary to elucidate the long-term refractive outcomes in such eyes. Secondly, the sample data were kept rather limited to make it possible to detect rare complications such as cataract formation in this study population. A large number of patients is required to assess the long-term incidence of cataract formation, especially in these presbyopic subjects undergoing hole ICL implantation. Thirdly, we did not measure the amplitude of accommodation in this study. Although this measurement is known to be time-consuming and not very reproducible, it may provide us with further information.

In summary, our pilot study supports the view that intentional undercorrection by hole ICL implantation is clinically useful for acquiring overall good binocular visual performance at all distances (from near to far) in early presbyopic subjects without developing cataract. We believe that this new presbyopic approach may be one of the viable surgical options for early presbyopic subjects.

## Figures and Tables

**Table 1:** Preoperative demographics of the study population undergoing hole implantable collamer lens (hole ICL) implantation.

Characteristic	Mean ± standard deviation
Age (years)	45.0 ± 3.8 years (range, 40 to 53 years)
Gender (male : female)	10 : 11
Manifest spherical equivalent (D)	-7.37 ± 3.18 D (range, -2.25 to -14.75 D)
Manifest cylinder (D)	1.15 ± 1.36 D (range, 0.00 to 6.00 D)
LogMAR UDVA	1.30 ± 0.24 (range, 1.00 to 1.70)
LogMAR CDVA	-0.17 ± 0.07 (range, -0.30 to 0.00)
White-to-white distance (mm)	11.6 ± 0.4 mm (range, 11.0 to 12.3 mm)
Anterior chamber depth (mm)	3.04 ± 0.22 mm (range, 2.80 to 3.55 mm)

D=diopter, logMAR=logarithm of the minimal angle of resolution,

UDVA=uncorrected distance visual acuity, and CDVA=corrected distance visual acuity.
